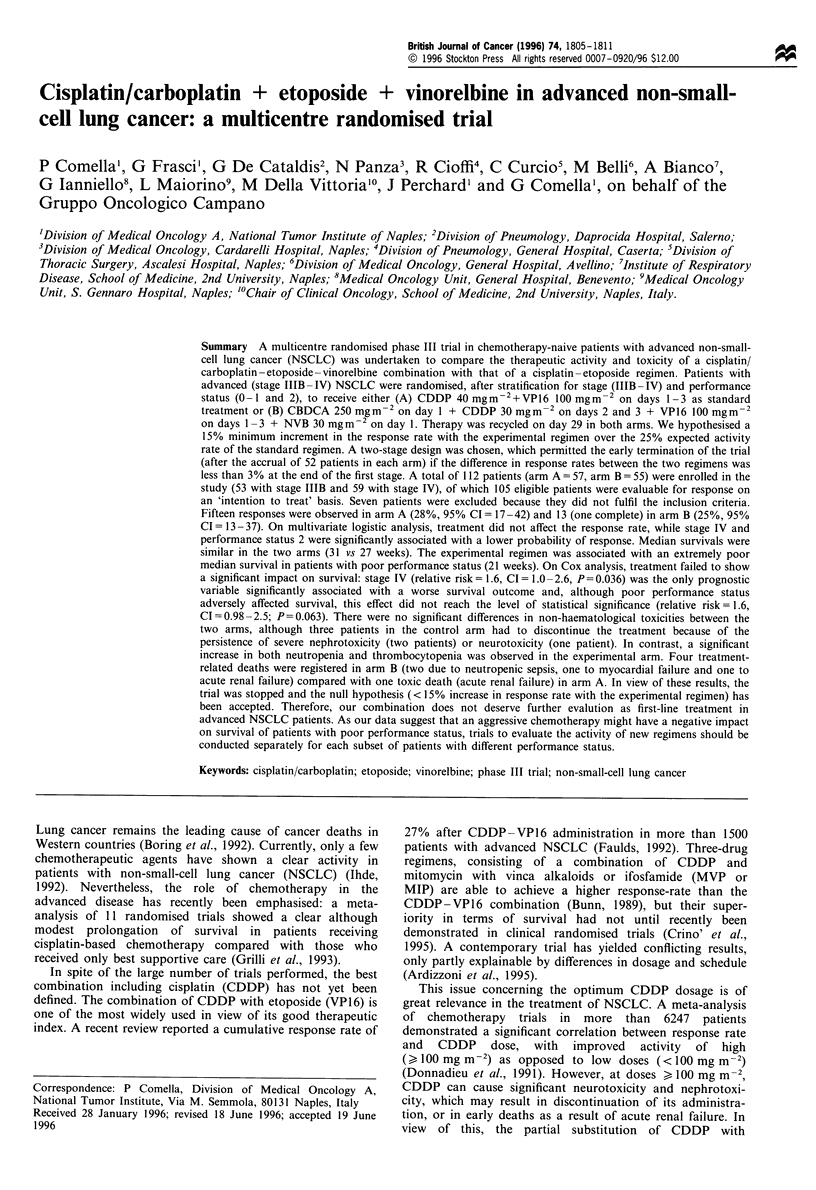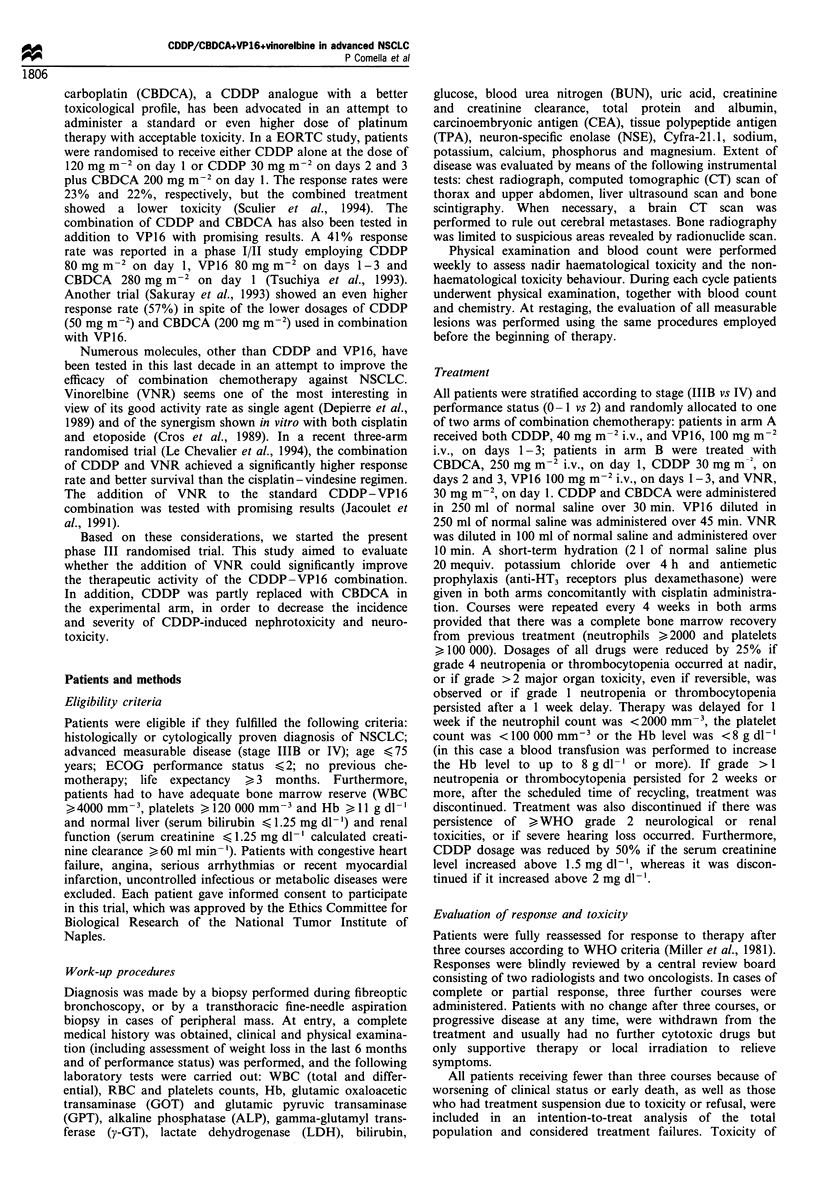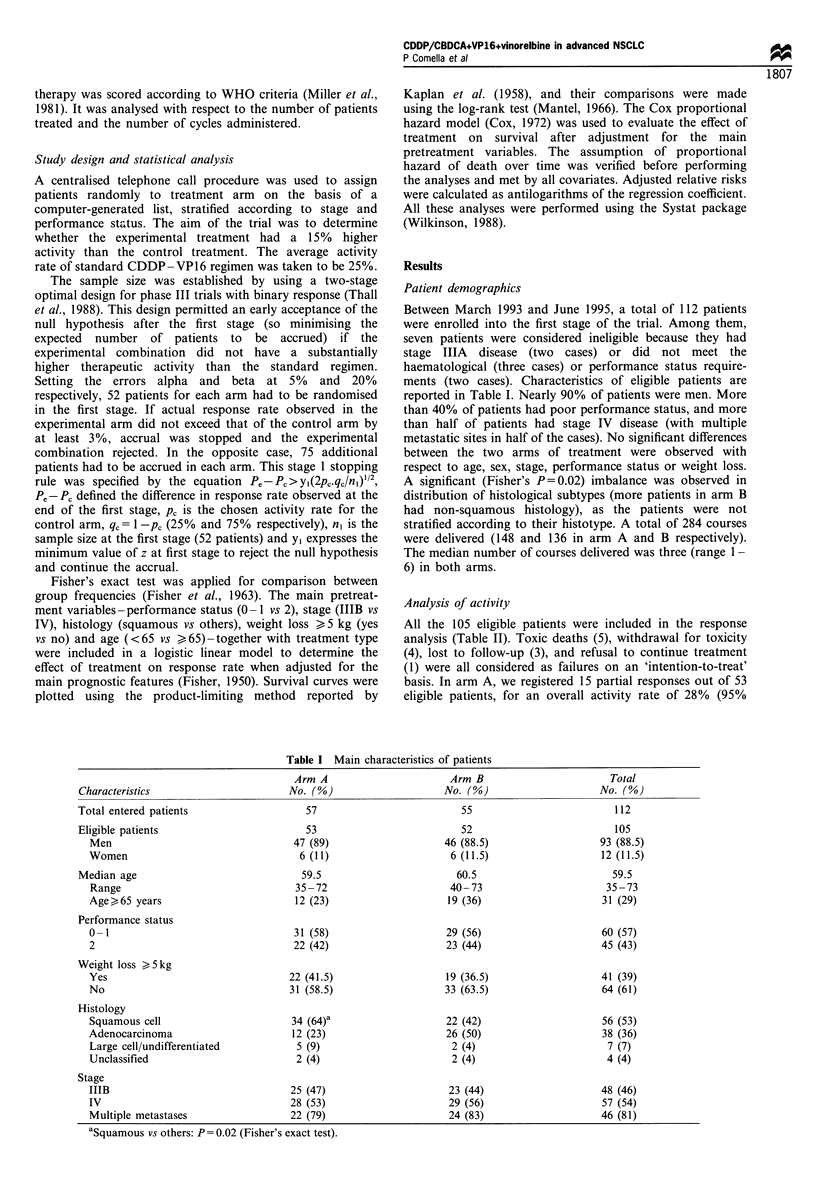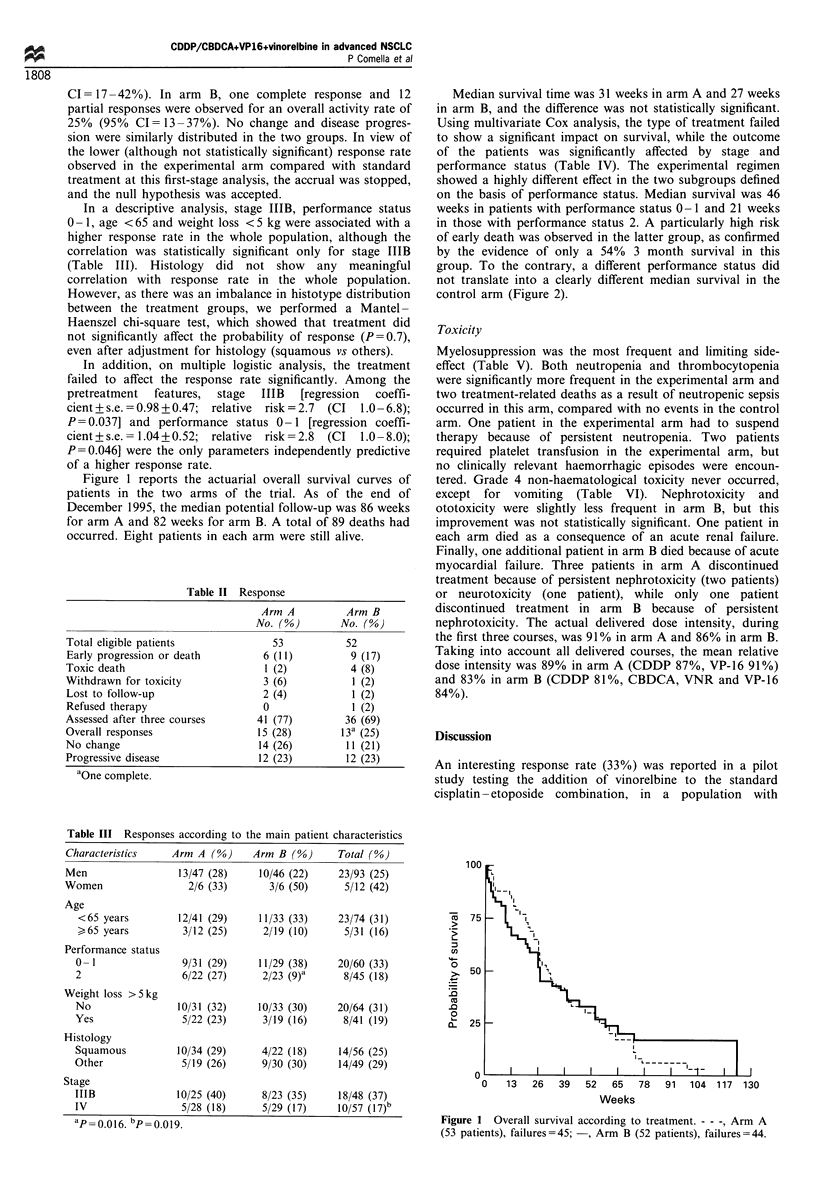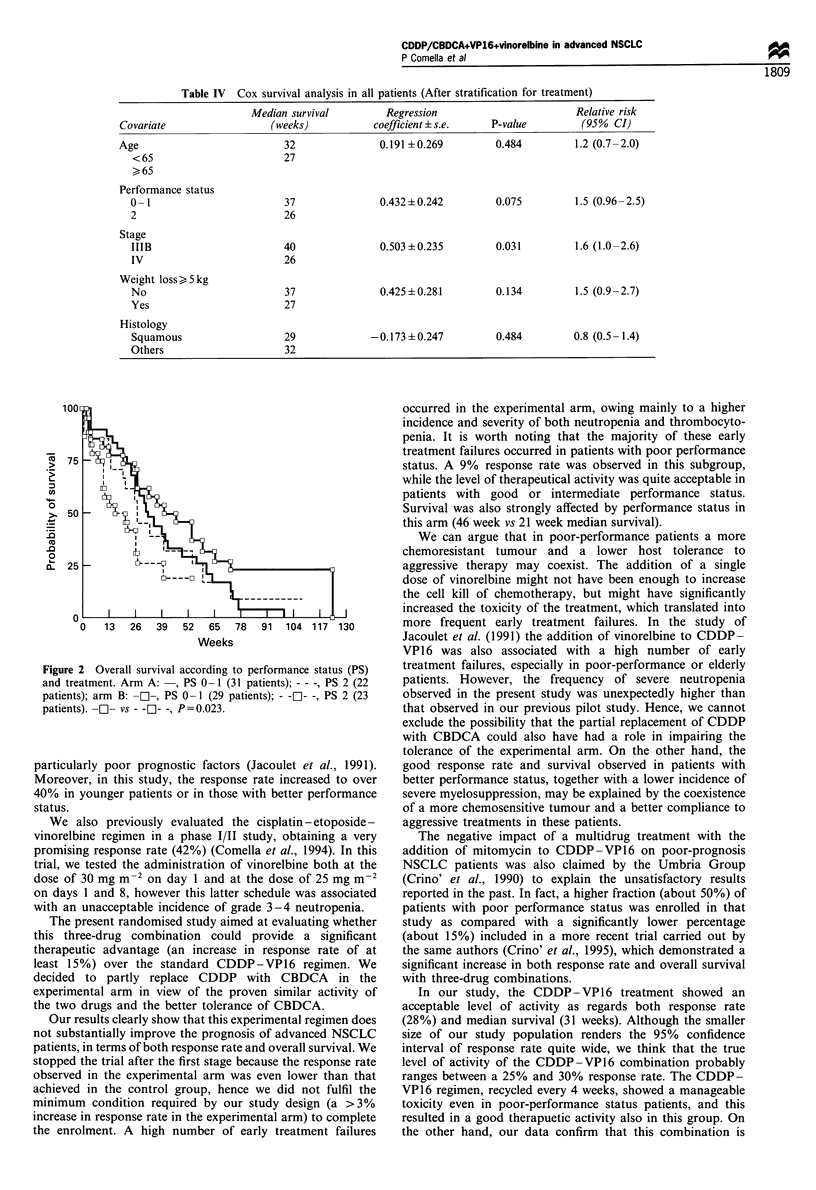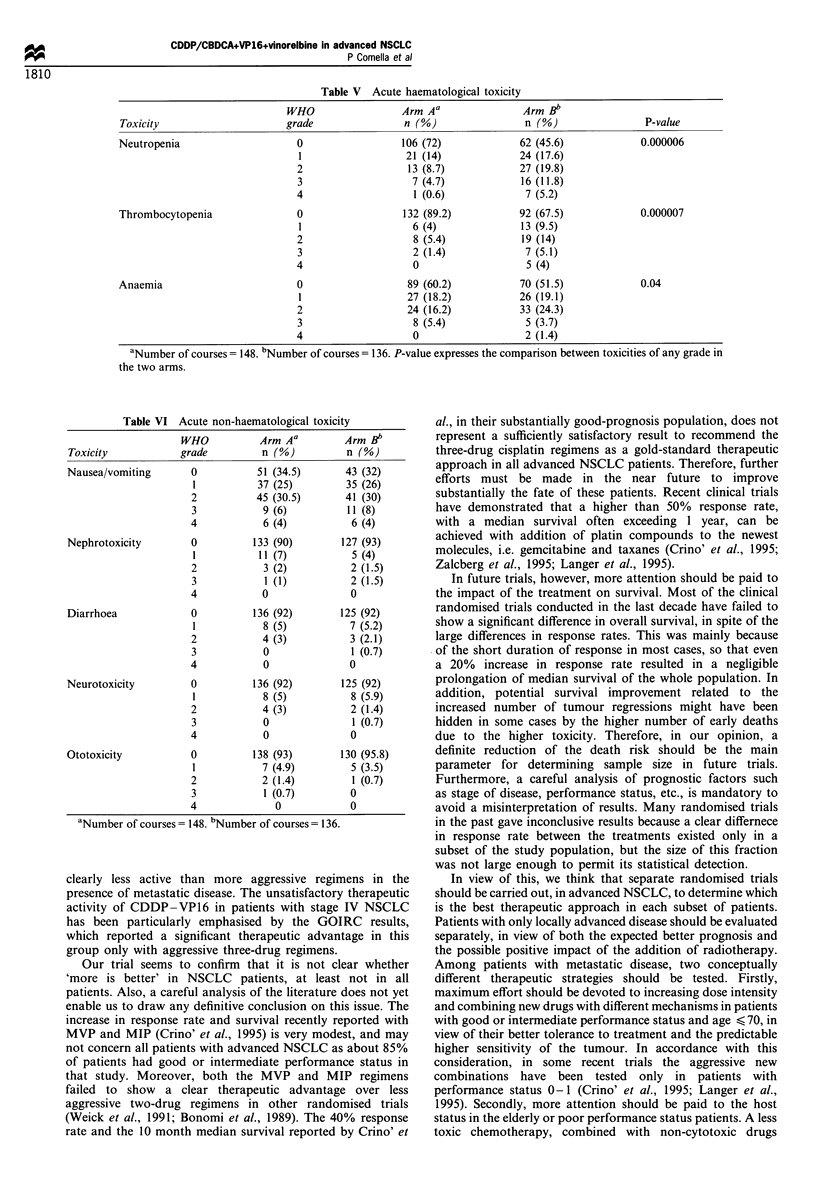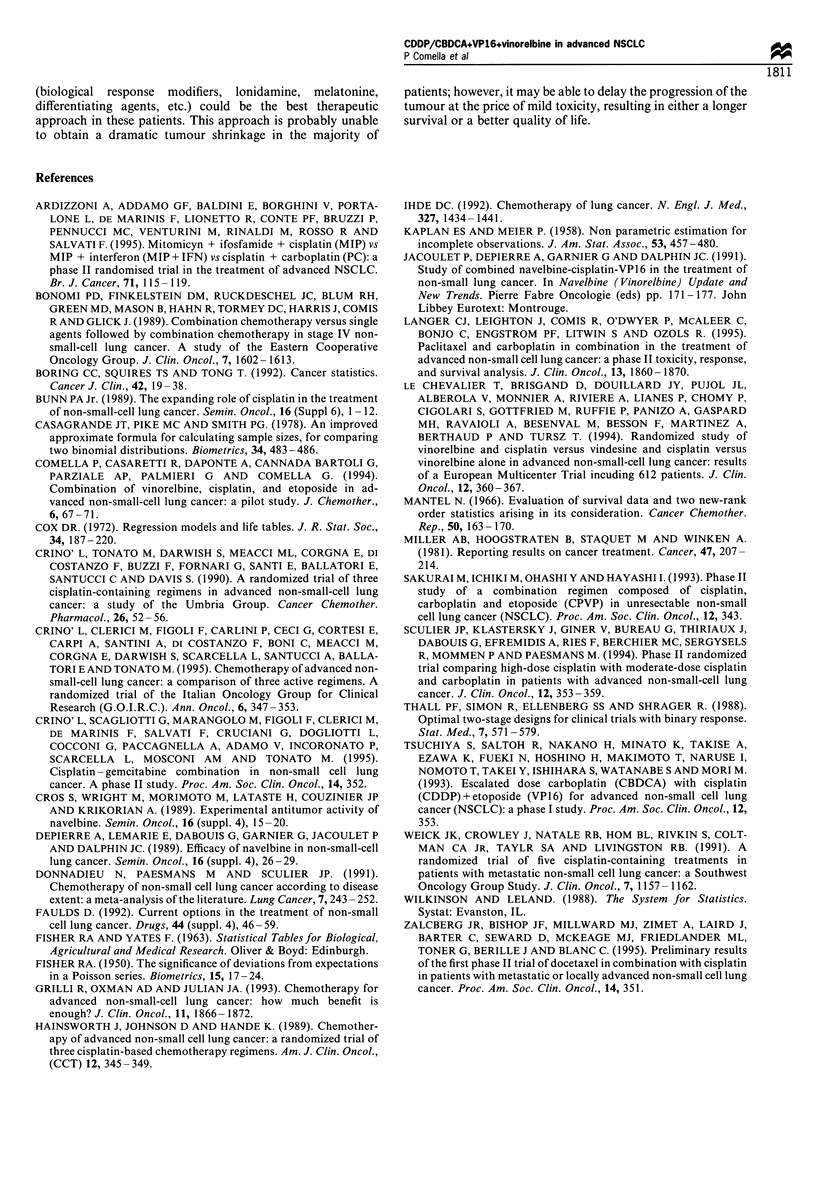# Cisplatin/carboplatin + etoposide + vinorelbine in advanced non-small-cell lung cancer: a multicentre randomised trial. Gruppo Oncologico Campano.

**DOI:** 10.1038/bjc.1996.634

**Published:** 1996-12

**Authors:** P. Comella, G. Frasci, G. De Cataldis, N. Panza, R. Cioffi, C. Curcio, M. Belli, A. Bianco, G. Ianniello, L. Maiorino, M. Della Vittoria, J. Perchard, G. Comella

**Affiliations:** Division of Medical Oncology A, National Tumor Institute of Naples, Italy.

## Abstract

A multicentre randomised phase III trial in chemotherapy-naive patients with advanced non-small-cell lung cancer (NSCLC) was undertaken to compare the therapeutic activity and toxicity of a cisplatin/carboplatin-etoposide-vinorelbine combination with that of a cisplatin-etoposide regimen. Patients with advanced (stage IIIB-IV) NSCLC were randomised, after stratification for stage (IIIB-IV) and performance status (0-1 and 2), to receive either (A) CDDP 40 mg m-2 + VP16 100 mg m-2 on days 1-3 as standard treatment or (B) CBDCA 250 mg m-2 on day 1 + CDDP 30 mg m-2 on days 2 and 3 + VP16 100 mg m-2 on days 1-3 + NVB 30 mg m-2 on day 1. Therapy was recycled on day 29 in both arms. We hypothesised a 15% minimum increment in the response rate with the experimental regimen over the 25% expected activity rate of the standard regimen. A two-stage design was chosen, which permitted the early termination of the trial (after the accrual of 52 patients in each arm) if the difference in response rates between the two regimens was less than 3% at the end of the first stage. A total of 112 patients (arm A = 57, arm B = 55) were enrolled in the study (53 with stage IIIB and 59 with stage IV), of which 105 eligible patients were evaluable for response on an "intention to treat' basis. Seven patients were excluded because they did not fulfil the inclusion criteria. Fifteen responses were observed in arm A (28%, 95% CI = 17-42) and 13 (one complete) in arm B (25%, 95% CI = 13-37). On multivariate logistic analysis, treatment did not affect the response rate, while stage IV and performance status 2 were significantly associated with a lower probability of response. Median survivals were similar in the two arms (31 vs 27 weeks). The experimental regimen was associated with an extremely poor median survival in patients with poor performance status (21 weeks). On Cox analysis, treatment failed to show a significant impact on survival: stage IV (relative risk = 1.6. CI = 1.0-2.6, P = 0.036) was the only prognostic variable significantly associated with a worse survival outcome and, although poor performance status adversely affected survival, this effect did not reach the level of statistical significance (relative risk = 1.6, CI = 0.98-2.5; P = 0.063). There were no significant differences in non-haematological toxicities between the two arms, although three patients in the control arm had to discontinue the treatment because of the persistence of severe nephrotoxicity (two patients) or neurotoxicity (one patient). In contrast, a significant increase in both neutropenia and thrombocytopenia was observed in the experimental arm. Four treatment-related deaths were registered in arm B (two due to neutropenic sepsis, one to myocardial failure and one to acute renal failure) compared with one toxic death (acute renal failure) in arm A. In view of these results, the trial was stopped and the null hypothesis (< 15% increase in response rate with the experimental regimen) has been accepted. Therefore, our combination does not deserve further evaluation as first-line treatment in advanced NSCLC patients. As our data suggest that an aggressive chemotherapy might have a negative impact on survival of patients with poor performance status, trials to evaluate the activity of new regimens should be conducted separately for each subset of patients with different performance status.


British Journal of Cancer (1996) 74, 1805-1811
© 1996 Stockton Press All rights reserved 0007-0920/96 $12.00

### Cisplatin/carboplatin + etoposide + vinorelbine in advanced non-small-cell lung cancer: a multicentre randomised trial

P Comella[1], G Frasci[1], G De Cataldis[2], N Panza[3], R Cioffi[4], C Curcio[5], M Belli[6], A Bianco[7], G Ianniello[8], L Maiorino[9], M Della Vittoria[10], J Perchard[1] and G Comella[1], on behalf of the Gruppo Oncologico Campano

[1]*Division of Medical Oncology A, National Tumor Institute of Naples;* [2]*Division of Pneumology, Daprocida Hospital, Salerno;* [3]*Division of Medical Oncology, Cardarelli Hospital, Naples;* [4]*Division of Pneumology, General Hospital, Caserta;* [5]*Division of Thoracic Surgery, Ascalesi Hospital, Naples;* [6]*Division of Medical Oncology, General Hospital, Avellino;* [7]*Institute of Respiratory Disease, School of Medicine, 2nd University, Naples;* [8]*Medical Oncology Unit, General Hospital, Benevento;* [9]*Medical Oncology Unit, S. Gennaro Hospital, Naples;* [10]*Chair of Clinical Oncology, School of Medicine, 2nd University, Naples, Italy.*

**Summary** A multicentre randomised phase III trial in chemotherapy-naive patients with advanced non-small-cell lung cancer (NSCLC) was undertaken to compare the therapeutic activity and toxicity of a cisplatin/carboplatin–etoposide–vinorelbine combination with that of a cisplatin–etoposide regimen. Patients with advanced (stage IIIB–IV) NSCLC were randomised, after stratification for stage (IIIB–IV) and performance status (0–1 and 2), to receive either (A) CDDP 40 mg $m^{-2}$ + VP16 100 mg $m^{-2}$ on days 1–3 as standard treatment or (B) CBDCA 250 mg $m^{-2}$ on day 1 + CDDP 30 mg $m^{-2}$ on days 2 and 3 + VP16 100 mg $m^{-2}$ on days 1–3 + NVB 30 mg $m^{-2}$ on day 1. Therapy was recycled on day 29 in both arms. We hypothesised a 15% minimum increment in the response rate with the experimental regimen over the 25% expected activity rate of the standard regimen. A two-stage design was chosen, which permitted the early termination of the trial (after the accrual of 52 patients in each arm) if the difference in response rates between the two regimens was less than 3% at the end of the first stage. A total of 112 patients (arm A = 57, arm B = 55) were enrolled in the study (53 with stage IIIB and 59 with stage IV), of which 105 eligible patients were evaluable for response on an 'intention to treat' basis. Seven patients were excluded because they did not fulfil the inclusion criteria. Fifteen responses were observed in arm A (28%, 95% CI = 17–42) and 13 (one complete) in arm B (25%, 95% CI = 13–37). On multivariate logistic analysis, treatment did not affect the response rate, while stage IV and performance status 2 were significantly associated with a lower probability of response. Median survivals were similar in the two arms (31 *vs* 27 weeks). The experimental regimen was associated with an extremely poor median survival in patients with poor performance status (21 weeks). On Cox analysis, treatment failed to show a significant impact on survival: stage IV (relative risk = 1.6, CI = 1.0–2.6, $P = 0.036$) was the only prognostic variable significantly associated with a worse survival outcome and, although poor performance status adversely affected survival, this effect did not reach the level of statistical significance (relative risk = 1.6, CI = 0.98–2.5; $P = 0.063$). There were no significant differences in non-haematological toxicities between the two arms, although three patients in the control arm had to discontinue the treatment because of the persistence of severe nephrotoxicity (two patients) or neurotoxicity (one patient). In contrast, a significant increase in both neutropenia and thrombocytopenia was observed in the experimental arm. Four treatment-related deaths were registered in arm B (two due to neutropenic sepsis, one to myocardial failure and one to acute renal failure) compared with one toxic death (acute renal failure) in arm A. In view of these results, the trial was stopped and the null hypothesis (< 15% increase in response rate with the experimental regimen) has been accepted. Therefore, our combination does not deserve further evalution as first-line treatment in advanced NSCLC patients. As our data suggest that an aggressive chemotherapy might have a negative impact on survival of patients with poor performance status, trials to evaluate the activity of new regimens should be conducted separately for each subset of patients with different performance status.

**Keywords:** cisplatin/carboplatin; etoposide; vinorelbine; phase III trial; non-small-cell lung cancer

Lung cancer remains the leading cause of cancer deaths in Western countries (Boring *et al.*, 1992). Currently, only a few chemotherapeutic agents have shown a clear activity in patients with non-small-cell lung cancer (NSCLC) (Ihde, 1992). Nevertheless, the role of chemotherapy in the advanced disease has recently been emphasised: a meta-analysis of 11 randomised trials showed a clear although modest prolongation of survival in patients receiving cisplatin-based chemotherapy compared with those who received only best supportive care (Grilli *et al.*, 1993).

In spite of the large number of trials performed, the best combination including cisplatin (CDDP) has not yet been defined. The combination of CDDP with etoposide (VP16) is one of the most widely used in view of its good therapeutic index. A recent review reported a cumulative response rate of 27% after CDDP–VP16 administration in more than 1500 patients with advanced NSCLC (Faulds, 1992). Three-drug regimens, consisting of a combination of CDDP and mitomycin with vinca alkaloids or ifosfamide (MVP or MIP) are able to achieve a higher response-rate than the CDDP–VP16 combination (Bunn, 1989), but their superiority in terms of survival had not until recently been demonstrated in clinical randomised trials (Crino' *et al.*, 1995). A contemporary trial has yielded conflicting results, only partly explainable by differences in dosage and schedule (Ardizzoni *et al.*, 1995).

This issue concerning the optimum CDDP dosage is of great relevance in the treatment of NSCLC. A meta-analysis of chemotherapy trials in more than 6247 patients demonstrated a significant correlation between response rate and CDDP dose, with improved activity of high (⩾100 mg $m^{-2}$) as opposed to low doses (< 100 mg $m^{-2}$) (Donnadieu *et al.*, 1991). However, at doses ⩾100 mg $m^{-2}$, CDDP can cause significant neurotoxicity and nephrotoxicity, which may result in discontinuation of its administration, or in early deaths as a result of acute renal failure. In view of this, the partial substitution of CDDP with

Correspondence: P Comella, Division of Medical Oncology A, National Tumor Institute, Via M. Semmola, 80131 Naples, Italy
Received 28 January 1996; revised 18 June 1996; accepted 19 June 1996

carboplatin (CBDCA), a CDDP analogue with a better toxicological profile, has been advocated in an attempt to administer a standard or even higher dose of platinum therapy with acceptable toxicity. In a EORTC study, patients were randomised to receive either CDDP alone at the dose of 120 mg $m^{-2}$ on day 1 or CDDP 30 mg $m^{-2}$ on days 2 and 3 plus CBDCA 200 mg $m^{-2}$ on day 1. The response rates were 23% and 22%, respectively, but the combined treatment showed a lower toxicity (Sculier *et al.*, 1994). The combination of CDDP and CBDCA has also been tested in addition to VP16 with promising results. A 41% response rate was reported in a phase I/II study employing CDDP 80 mg $m^{-2}$ on day 1, VP16 80 mg $m^{-2}$ on days 1–3 and CBDCA 280 mg $m^{-2}$ on day 1 (Tsuchiya *et al.*, 1993). Another trial (Sakuray *et al.*, 1993) showed an even higher response rate (57%) in spite of the lower dosages of CDDP (50 mg $m^{-2}$) and CBDCA (200 mg $m^{-2}$) used in combination with VP16.

Numerous molecules, other than CDDP and VP16, have been tested in this last decade in an attempt to improve the efficacy of combination chemotherapy against NSCLC. Vinorelbine (VNR) seems one of the most interesting in view of its good activity rate as single agent (Depierre *et al.*, 1989) and of the synergism shown *in vitro* with both cisplatin and etoposide (Cros *et al.*, 1989). In a recent three-arm randomised trial (Le Chevalier *et al.*, 1994), the combination of CDDP and VNR achieved a significantly higher response rate and better survival than the cisplatin–vindesine regimen. The addition of VNR to the standard CDDP–VP16 combination was tested with promising results (Jacoulet *et al.*, 1991).

Based on these considerations, we started the present phase III randomised trial. This study aimed to evaluate whether the addition of VNR could significantly improve the therapeutic activity of the CDDP–VP16 combination. In addition, CDDP was partly replaced with CBDCA in the experimental arm, in order to decrease the incidence and severity of CDDP-induced nephrotoxicity and neurotoxicity.

### Patients and methods

### *Eligibility criteria*

Patients were eligible if they fulfilled the following criteria: histologically or cytologically proven diagnosis of NSCLC; advanced measurable disease (stage IIIB or IV); age $\leqslant 75$ years; ECOG performance status $\leqslant 2$; no previous chemotherapy; life expectancy $\geqslant 3$ months. Furthermore, patients had to have adequate bone marrow reserve (WBC $\geqslant 4000$ $mm^{-3}$, platelets $\geqslant 120\,000$ $mm^{-3}$ and Hb $\geqslant 11$ g $dl^{-1}$ and normal liver (serum bilirubin $\leqslant 1.25$ mg $dl^{-1}$) and renal function (serum creatinine $\leqslant 1.25$ mg $dl^{-1}$ calculated creatinine clearance $\geqslant 60$ ml $min^{-1}$). Patients with congestive heart failure, angina, serious arrhythmias or recent myocardial infarction, uncontrolled infectious or metabolic diseases were excluded. Each patient gave informed consent to participate in this trial, which was approved by the Ethics Committee for Biological Research of the National Tumor Institute of Naples.

### *Work-up procedures*

Diagnosis was made by a biopsy performed during fibreoptic bronchoscopy, or by a transthoracic fine-needle aspiration biopsy in cases of peripheral mass. At entry, a complete medical history was obtained, clinical and physical examination (including assessment of weight loss in the last 6 months and of performance status) was performed, and the following laboratory tests were carried out: WBC (total and differential), RBC and platelets counts, Hb, glutamic oxaloacetic transaminase (GOT) and glutamic pyruvic transaminase (GPT), alkaline phosphatase (ALP), gamma-glutamyl transferase ($\gamma$-GT), lactate dehydrogenase (LDH), bilirubin, glucose, blood urea nitrogen (BUN), uric acid, creatinine and creatinine clearance, total protein and albumin, carcinoembryonic antigen (CEA), tissue polypeptide antigen (TPA), neuron-specific enolase (NSE), Cyfra-21.1, sodium, potassium, calcium, phosphorus and magnesium. Extent of disease was evaluated by means of the following instrumental tests: chest radiograph, computed tomographic (CT) scan of thorax and upper abdomen, liver ultrasound scan and bone scintigraphy. When necessary, a brain CT scan was performed to rule out cerebral metastases. Bone radiography was limited to suspicious areas revealed by radionuclide scan.

Physical examination and blood count were performed weekly to assess nadir haematological toxicity and the non-haematological toxicity behaviour. During each cycle patients underwent physical examination, together with blood count and chemistry. At restaging, the evaluation of all measurable lesions was performed using the same procedures employed before the beginning of therapy.

### *Treatment*

All patients were stratified according to stage (IIIB *vs* IV) and performance status (0–1 *vs* 2) and randomly allocated to one of two arms of combination chemotherapy: patients in arm A received both CDDP, 40 mg $m^{-2}$ i.v., and VP16, 100 mg $m^{-2}$ i.v., on days 1–3; patients in arm B were treated with CBDCA, 250 mg $m^{-2}$ i.v., on day 1, CDDP 30 mg $m^{-2}$, on days 2 and 3, VP16 100 mg $m^{-2}$ i.v., on days 1–3, and VNR, 30 mg $m^{-2}$, on day 1. CDDP and CBDCA were administered in 250 ml of normal saline over 30 min. VP16 diluted in 250 ml of normal saline was administered over 45 min. VNR was diluted in 100 ml of normal saline and administered over 10 min. A short-term hydration (2 l of normal saline plus 20 mequiv. potassium chloride over 4 h and antiemetic prophylaxis (anti-$HT_3$ receptors plus dexamethasone) were given in both arms concomitantly with cisplatin administration. Courses were repeated every 4 weeks in both arms provided that there was a complete bone marrow recovery from previous treatment (neutrophils $\geqslant 2000$ and platelets $\geqslant 100\,000$). Dosages of all drugs were reduced by 25% if grade 4 neutropenia or thrombocytopenia occurred at nadir, or if grade $>2$ major organ toxicity, even if reversible, was observed or if grade 1 neutropenia or thrombocytopenia persisted after a 1 week delay. Therapy was delayed for 1 week if the neutrophil count was $<2000$ $mm^{-3}$, the platelet count was $<100\,000$ $mm^{-3}$ or the Hb level was $<8$ g $dl^{-1}$ (in this case a blood transfusion was performed to increase the Hb level to up to 8 g $dl^{-1}$ or more). If grade $>1$ neutropenia or thrombocytopenia persisted for 2 weeks or more, after the scheduled time of recycling, treatment was discontinued. Treatment was also discontinued if there was persistence of $\geqslant$WHO grade 2 neurological or renal toxicities, or if severe hearing loss occurred. Furthermore, CDDP dosage was reduced by 50% if the serum creatinine level increased above 1.5 mg $dl^{-1}$, whereas it was discontinued if it increased above 2 mg $dl^{-1}$.

### *Evaluation of response and toxicity*

Patients were fully reassessed for response to therapy after three courses according to WHO criteria (Miller *et al.*, 1981). Responses were blindly reviewed by a central review board consisting of two radiologists and two oncologists. In cases of complete or partial response, three further courses were administered. Patients with no change after three courses, or progressive disease at any time, were withdrawn from the treatment and usually had no further cytotoxic drugs but only supportive therapy or local irradiation to relieve symptoms.

All patients receiving fewer than three courses because of worsening of clinical status or early death, as well as those who had treatment suspension due to toxicity or refusal, were included in an intention-to-treat analysis of the total population and considered treatment failures. Toxicity of therapy was scored according to WHO criteria (Miller *et al.*, 1981). It was analysed with respect to the number of patients treated and the number of cycles administered.

*Study design and statistical analysis*

A centralised telephone call procedure was used to assign patients randomly to treatment arm on the basis of a computer-generated list, stratified according to stage and performance status. The aim of the trial was to determine whether the experimental treatment had a 15% higher activity than the control treatment. The average activity rate of standard CDDP–VP16 regimen was taken to be 25%.

The sample size was established by using a two-stage optimal design for phase III trials with binary response (Thall *et al.*, 1988). This design permitted an early acceptance of the null hypothesis after the first stage (so minimising the expected number of patients to be accrued) if the experimental combination did not have a substantially higher therapeutic activity than the standard regimen. Setting the errors alpha and beta at 5% and 20% respectively, 52 patients for each arm had to be randomised in the first stage. If actual response rate observed in the experimental arm did not exceed that of the control arm by at least 3%, accrual was stopped and the experimental combination rejected. In the opposite case, 75 additional patients had to be accrued in each arm. This stage 1 stopping rule was specified by the equation $P_e - P_c > y_1(2p_c{\cdot}q_c/n_1)^{1/2}$, $P_e - P_c$ defined the difference in response rate observed at the end of the first stage, $p_c$ is the chosen activity rate for the control arm, $q_c = 1 - p_c$ (25% and 75% respectively), $n_1$ is the sample size at the first stage (52 patients) and $y_1$ expresses the minimum value of $z$ at first stage to reject the null hypothesis and continue the accrual.

Fisher's exact test was applied for comparison between group frequencies (Fisher *et al.*, 1963). The main pretreatment variables–performance status (0–1 *vs* 2), stage (IIIB *vs* IV), histology (squamous *vs* others), weight loss ⩾5 kg (yes *vs* no) and age (<65 *vs* ⩾65)–together with treatment type were included in a logistic linear model to determine the effect of treatment on response rate when adjusted for the main prognostic features (Fisher, 1950). Survival curves were plotted using the product-limiting method reported by Kaplan *et al.* (1958), and their comparisons were made using the log-rank test (Mantel, 1966). The Cox proportional hazard model (Cox, 1972) was used to evaluate the effect of treatment on survival after adjustment for the main pretreatment variables. The assumption of proportional hazard of death over time was verified before performing the analyses and met by all covariates. Adjusted relative risks were calculated as antilogarithms of the regression coefficient. All these analyses were performed using the Systat package (Wilkinson, 1988).

### Results

*Patient demographics*

Between March 1993 and June 1995, a total of 112 patients were enrolled into the first stage of the trial. Among them, seven patients were considered ineligible because they had stage IIIA disease (two cases) or did not meet the haematological (three cases) or performance status requirements (two cases). Characteristics of eligible patients are reported in Table I. Nearly 90% of patients were men. More than 40% of patients had poor performance status, and more than half of patients had stage IV disease (with multiple metastatic sites in half of the cases). No significant differences between the two arms of treatment were observed with respect to age, sex, stage, performance status or weight loss. A significant (Fisher's $P = 0.02$) imbalance was observed in distribution of histological subtypes (more patients in arm B had non-squamous histology), as the patients were not stratified according to their histotype. A total of 284 courses were delivered (148 and 136 in arm A and B respectively). The median number of courses delivered was three (range 1–6) in both arms.

*Analysis of activity*

All the 105 eligible patients were included in the response analysis (Table II). Toxic deaths (5), withdrawal for toxicity (4), lost to follow-up (3), and refusal to continue treatment (1) were all considered as failures on an 'intention-to-treat' basis. In arm A, we registered 15 partial responses out of 53 eligible patients, for an overall activity rate of 28% (95%

**Table I** Main characteristics of patients

| *Characteristics* | *Arm A* No. (%) | *Arm B* No. (%) | *Total* No. (%) |
|---|---|---|---|
| Total entered patients | 57 | 55 | 112 |
| Eligible patients | 53 | 52 | 105 |
| Men | 47 (89) | 46 (88.5) | 93 (88.5) |
| Women | 6 (11) | 6 (11.5) | 12 (11.5) |
| Median age | 59.5 | 60.5 | 59.5 |
| Range | 35–72 | 40–73 | 35–73 |
| Age⩾65 years | 12 (23) | 19 (36) | 31 (29) |
| Performance status | | | |
| 0–1 | 31 (58) | 29 (56) | 60 (57) |
| 2 | 22 (42) | 23 (44) | 45 (43) |
| Weight loss ⩾5 kg | | | |
| Yes | 22 (41.5) | 19 (36.5) | 41 (39) |
| No | 31 (58.5) | 33 (63.5) | 64 (61) |
| Histology | | | |
| Squamous cell | 34 (64)[a] | 22 (42) | 56 (53) |
| Adenocarcinoma | 12 (23) | 26 (50) | 38 (36) |
| Large cell/undifferentiated | 5 (9) | 2 (4) | 7 (7) |
| Unclassified | 2 (4) | 2 (4) | 4 (4) |
| Stage | | | |
| IIIB | 25 (47) | 23 (44) | 48 (46) |
| IV | 28 (53) | 29 (56) | 57 (54) |
| Multiple metastases | 22 (79) | 24 (83) | 46 (81) |

[a]Squamous *vs* others: $P = 0.02$ (Fisher's exact test).

CI = 17–42%). In arm B, one complete response and 12 partial responses were observed for an overall activity rate of 25% (95% CI = 13–37%). No change and disease progression were similarly distributed in the two groups. In view of the lower (although not statistically significant) response rate observed in the experimental arm compared with standard treatment at this first-stage analysis, the accrual was stopped, and the null hypothesis was accepted.

In a descriptive analysis, stage IIIB, performance status 0–1, age <65 and weight loss <5 kg were associated with a higher response rate in the whole population, although the correlation was statistically significant only for stage IIIB (Table III). Histology did not show any meaningful correlation with response rate in the whole population. However, as there was an imbalance in histotype distribution between the treatment groups, we performed a Mantel–Haenszel chi-square test, which showed that treatment did not significantly affect the probability of response ($P = 0.7$), even after adjustment for histology (squamous *vs* others).

In addition, on multiple logistic analysis, the treatment failed to affect the response rate significantly. Among the pretreatment features, stage IIIB [regression coefficient ± s.e. = 0.98 ± 0.47; relative risk = 2.7 (CI 1.0–6.8); $P = 0.037$] and performance status 0–1 [regression coefficient ± s.e. = 1.04 ± 0.52; relative risk = 2.8 (CI 1.0–8.0); $P = 0.046$] were the only parameters independently predictive of a higher response rate.

Figure 1 reports the actuarial overall survival curves of patients in the two arms of the trial. As of the end of December 1995, the median potential follow-up was 86 weeks for arm A and 82 weeks for arm B. A total of 89 deaths had occurred. Eight patients in each arm were still alive.

**Table II** Response

| | *Arm A* No. (%) | *Arm B* No. (%) |
|---|---|---|
| Total eligible patients | 53 | 52 |
| Early progression or death | 6 (11) | 9 (17) |
| Toxic death | 1 (2) | 4 (8) |
| Withdrawn for toxicity | 3 (6) | 1 (2) |
| Lost to follow-up | 2 (4) | 1 (2) |
| Refused therapy | 0 | 1 (2) |
| Assessed after three courses | 41 (77) | 36 (69) |
| Overall responses | 15 (28) | 13[a] (25) |
| No change | 14 (26) | 11 (21) |
| Progressive disease | 12 (23) | 12 (23) |

[a]One complete.

**Table III** Responses according to the main patient characteristics

| *Characteristics* | *Arm A (%)* | *Arm B (%)* | *Total (%)* |
|---|---|---|---|
| Men | 13/47 (28) | 10/46 (22) | 23/93 (25) |
| Women | 2/6 (33) | 3/6 (50) | 5/12 (42) |
| Age | | | |
| <65 years | 12/41 (29) | 11/33 (33) | 23/74 (31) |
| ⩾65 years | 3/12 (25) | 2/19 (10) | 5/31 (16) |
| Performance status | | | |
| 0–1 | 9/31 (29) | 11/29 (38) | 20/60 (33) |
| 2 | 6/22 (27) | 2/23 (9)[a] | 8/45 (18) |
| Weight loss >5 kg | | | |
| No | 10/31 (32) | 10/33 (30) | 20/64 (31) |
| Yes | 5/22 (23) | 3/19 (16) | 8/41 (19) |
| Histology | | | |
| Squamous | 10/34 (29) | 4/22 (18) | 14/56 (25) |
| Other | 5/19 (26) | 9/30 (30) | 14/49 (29) |
| Stage | | | |
| IIIB | 10/25 (40) | 8/23 (35) | 18/48 (37) |
| IV | 5/28 (18) | 5/29 (17) | 10/57 (17)[b] |

[a]$P = 0.016$. [b]$P = 0.019$.

Median survival time was 31 weeks in arm A and 27 weeks in arm B, and the difference was not statistically significant. Using multivariate Cox analysis, the type of treatment failed to show a significant impact on survival, while the outcome of the patients was significantly affected by stage and performance status (Table IV). The experimental regimen showed a highly different effect in the two subgroups defined on the basis of performance status. Median survival was 46 weeks in patients with performance status 0–1 and 21 weeks in those with performance status 2. A particularly high risk of early death was observed in the latter group, as confirmed by the evidence of only a 54% 3 month survival in this group. To the contrary, a different performance status did not translate into a clearly different median survival in the control arm (Figure 2).

*Toxicity*

Myelosuppression was the most frequent and limiting side-effect (Table V). Both neutropenia and thrombocytopenia were significantly more frequent in the experimental arm and two treatment-related deaths as a result of neutropenic sepsis occurred in this arm, compared with no events in the control arm. One patient in the experimental arm had to suspend therapy because of persistent neutropenia. Two patients required platelet transfusion in the experimental arm, but no clinically relevant haemorrhagic episodes were encountered. Grade 4 non-haematological toxicity never occurred, except for vomiting (Table VI). Nephrotoxicity and ototoxicity were slightly less frequent in arm B, but this improvement was not statistically significant. One patient in each arm died as a consequence of an acute renal failure. Finally, one additional patient in arm B died because of acute myocardial failure. Three patients in arm A discontinued treatment because of persistent nephrotoxicity (two patients) or neurotoxicity (one patient), while only one patient discontinued treatment in arm B because of persistent nephrotoxicity. The actual delivered dose intensity, during the first three courses, was 91% in arm A and 86% in arm B. Taking into account all delivered courses, the mean relative dose intensity was 89% in arm A (CDDP 87%, VP-16 91%) and 83% in arm B (CDDP 81%, CBDCA, VNR and VP-16 84%).

### Discussion

An interesting response rate (33%) was reported in a pilot study testing the addition of vinorelbine to the standard cisplatin–etoposide combination, in a population with

**Figure 1** Overall survival according to treatment. - - -, Arm A (53 patients), failures = 45; —, Arm B (52 patients), failures = 44.

**Table IV** Cox survival analysis in all patients (After stratification for treatment)

| *Covariate* | *Median survival (weeks)* | *Regression coefficient ± s.e.* | P-*value* | *Relative risk (95% CI)* |
|---|---|---|---|---|
| Age | 32 | 0.191 ± 0.269 | 0.484 | 1.2 (0.7–2.0) |
| <65 | 27 | | | |
| ⩾65 | | | | |
| Performance status | | | | |
| 0–1 | 37 | 0.432 ± 0.242 | 0.075 | 1.5 (0.96–2.5) |
| 2 | 26 | | | |
| Stage | | | | |
| IIIB | 40 | 0.503 ± 0.235 | 0.031 | 1.6 (1.0–2.6) |
| IV | 26 | | | |
| Weight loss ⩾ 5 kg | | | | |
| No | 37 | 0.425 ± 0.281 | 0.134 | 1.5 (0.9–2.7) |
| Yes | 27 | | | |
| Histology | | | | |
| Squamous | 29 | −0.173 ± 0.247 | 0.484 | 0.8 (0.5–1.4) |
| Others | 32 | | | |

**Figure 2** Overall survival according to performance status (PS) and treatment. Arm A: —, PS 0–1 (31 patients); - - -, PS 2 (22 patients); arm B: –□–, PS 0–1 (29 patients); - -□- -, PS 2 (23 patients). –□– vs - -□- -, $P=0.023$.

particularly poor prognostic factors (Jacoulet *et al.*, 1991). Moreover, in this study, the response rate increased to over 40% in younger patients or in those with better performance status.

We also previously evaluated the cisplatin–etoposide–vinorelbine regimen in a phase I/II study, obtaining a very promising response rate (42%) (Comella *et al.*, 1994). In this trial, we tested the administration of vinorelbine both at the dose of 30 mg $m^{-2}$ on day 1 and at the dose of 25 mg $m^{-2}$ on days 1 and 8, however this latter schedule was associated with an unacceptable incidence of grade 3–4 neutropenia.

The present randomised study aimed at evaluating whether this three-drug combination could provide a significant therapeutic advantage (an increase in response rate of at least 15%) over the standard CDDP–VP16 regimen. We decided to partly replace CDDP with CBDCA in the experimental arm in view of the proven similar activity of the two drugs and the better tolerance of CBDCA.

Our results clearly show that this experimental regimen does not substantially improve the prognosis of advanced NSCLC patients, in terms of both response rate and overall survival. We stopped the trial after the first stage because the response rate observed in the experimental arm was even lower than that achieved in the control group, hence we did not fulfil the minimum condition required by our study design (a >3% increase in response rate in the experimental arm) to complete the enrolment. A high number of early treatment failures occurred in the experimental arm, owing mainly to a higher incidence and severity of both neutropenia and thrombocytopenia. It is worth noting that the majority of these early treatment failures occurred in patients with poor performance status. A 9% response rate was observed in this subgroup, while the level of therapeutical activity was quite acceptable in patients with good or intermediate performance status. Survival was also strongly affected by performance status in this arm (46 week *vs* 21 week median survival).

We can argue that in poor-performance patients a more chemoresistant tumour and a lower host tolerance to aggressive therapy may coexist. The addition of a single dose of vinorelbine might not have been enough to increase the cell kill of chemotherapy, but might have significantly increased the toxicity of the treatment, which translated into more frequent early treatment failures. In the study of Jacoulet *et al.* (1991) the addition of vinorelbine to CDDP–VP16 was also associated with a high number of early treatment failures, especially in poor-performance or elderly patients. However, the frequency of severe neutropenia observed in the present study was unexpectedly higher than that observed in our previous pilot study. Hence, we cannot exclude the possibility that the partial replacement of CDDP with CBDCA could also have had a role in impairing the tolerance of the experimental arm. On the other hand, the good response rate and survival observed in patients with better performance status, together with a lower incidence of severe myelosuppression, may be explained by the coexistence of a more chemosensitive tumour and a better compliance to aggressive treatments in these patients.

The negative impact of a multidrug treatment with the addition of mitomycin to CDDP–VP16 on poor-prognosis NSCLC patients was also claimed by the Umbria Group (Crino' *et al.*, 1990) to explain the unsatisfactory results reported in the past. In fact, a higher fraction (about 50%) of patients with poor performance status was enrolled in that study as compared with a significantly lower percentage (about 15%) included in a more recent trial carried out by the same authors (Crino' *et al.*, 1995), which demonstrated a significant increase in both response rate and overall survival with three-drug combinations.

In our study, the CDDP–VP16 treatment showed an acceptable level of activity as regards both response rate (28%) and median survival (31 weeks). Although the smaller size of our study population renders the 95% confidence interval of response rate quite wide, we think that the true level of activity of the CDDP–VP16 combination probably ranges between a 25% and 30% response rate. The CDDP–VP16 regimen, recycled every 4 weeks, showed a manageable toxicity even in poor-performance status patients, and this resulted in a good therapeutic activity also in this group. On the other hand, our data confirm that this combination is

**Table V** Acute haematological toxicity

| Toxicity | WHO grade | Arm A[a] n (%) | Arm B[b] n (%) | P-value |
|---|---|---|---|---|
| Neutropenia | 0 | 106 (72) | 62 (45.6) | 0.000006 |
| | 1 | 21 (14) | 24 (17.6) | |
| | 2 | 13 (8.7) | 27 (19.8) | |
| | 3 | 7 (4.7) | 16 (11.8) | |
| | 4 | 1 (0.6) | 7 (5.2) | |
| Thrombocytopenia | 0 | 132 (89.2) | 92 (67.5) | 0.000007 |
| | 1 | 6 (4) | 13 (9.5) | |
| | 2 | 8 (5.4) | 19 (14) | |
| | 3 | 2 (1.4) | 7 (5.1) | |
| | 4 | 0 | 5 (4) | |
| Anaemia | 0 | 89 (60.2) | 70 (51.5) | 0.04 |
| | 1 | 27 (18.2) | 26 (19.1) | |
| | 2 | 24 (16.2) | 33 (24.3) | |
| | 3 | 8 (5.4) | 5 (3.7) | |
| | 4 | 0 | 2 (1.4) | |

[a]Number of courses = 148. [b]Number of courses = 136. *P*-value expresses the comparison between toxicities of any grade in the two arms.

**Table VI** Acute non-haematological toxicity

| Toxicity | WHO grade | Arm A[a] n (%) | Arm B[b] n (%) |
|---|---|---|---|
| Nausea/vomiting | 0 | 51 (34.5) | 43 (32) |
| | 1 | 37 (25) | 35 (26) |
| | 2 | 45 (30.5) | 41 (30) |
| | 3 | 9 (6) | 11 (8) |
| | 4 | 6 (4) | 6 (4) |
| Nephrotoxicity | 0 | 133 (90) | 127 (93) |
| | 1 | 11 (7) | 5 (4) |
| | 2 | 3 (2) | 2 (1.5) |
| | 3 | 1 (1) | 2 (1.5) |
| | 4 | 0 | 0 |
| Diarrhoea | 0 | 136 (92) | 125 (92) |
| | 1 | 8 (5) | 7 (5.2) |
| | 2 | 4 (3) | 3 (2.1) |
| | 3 | 0 | 1 (0.7) |
| | 4 | 0 | 0 |
| Neurotoxicity | 0 | 136 (92) | 125 (92) |
| | 1 | 8 (5) | 8 (5.9) |
| | 2 | 4 (3) | 2 (1.4) |
| | 3 | 0 | 1 (0.7) |
| | 4 | 0 | 0 |
| Ototoxicity | 0 | 138 (93) | 130 (95.8) |
| | 1 | 7 (4.9) | 5 (3.5) |
| | 2 | 2 (1.4) | 1 (0.7) |
| | 3 | 1 (0.7) | 0 |
| | 4 | 0 | 0 |

[a]Number of courses = 148. [b]Number of courses = 136.

clearly less active than more aggressive regimens in the presence of metastatic disease. The unsatisfactory therapeutic activity of CDDP-VP16 in patients with stage IV NSCLC has been particularly emphasised by the GOIRC results, which reported a significant therapeutic advantage in this group only with aggressive three-drug regimens.

Our trial seems to confirm that it is not clear whether 'more is better' in NSCLC patients, at least not in all patients. Also, a careful analysis of the literature does not yet enable us to draw any definitive conclusion on this issue. The increase in response rate and survival recently reported with MVP and MIP (Crino' *et al.*, 1995) is very modest, and may not concern all patients with advanced NSCLC as about 85% of patients had good or intermediate performance status in that study. Moreover, both the MVP and MIP regimens failed to show a clear therapeutic advantage over less aggressive two-drug regimens in other randomised trials (Weick *et al.*, 1991; Bonomi *et al.*, 1989). The 40% response rate and the 10 month median survival reported by Crino' *et al.*, in their substantially good-prognosis population, does not represent a sufficiently satisfactory result to recommend the three-drug cisplatin regimens as a gold-standard therapeutic approach in all advanced NSCLC patients. Therefore, further efforts must be made in the near future to improve substantially the fate of these patients. Recent clinical trials have demonstrated that a higher than 50% response rate, with a median survival often exceeding 1 year, can be achieved with addition of platin compounds to the newest molecules, i.e. gemcitabine and taxanes (Crino' *et al.*, 1995; Zalcberg *et al.*, 1995; Langer *et al.*, 1995).

In future trials, however, more attention should be paid to the impact of the treatment on survival. Most of the clinical randomised trials conducted in the last decade have failed to show a significant difference in overall survival, in spite of the large differences in response rates. This was mainly because of the short duration of response in most cases, so that even a 20% increase in response rate resulted in a negligible prolongation of median survival of the whole population. In addition, potential survival improvement related to the increased number of tumour regressions might have been hidden in some cases by the higher number of early deaths due to the higher toxicity. Therefore, in our opinion, a definite reduction of the death risk should be the main parameter for determining sample size in future trials. Furthermore, a careful analysis of prognostic factors such as stage of disease, performance status, etc., is mandatory to avoid a misinterpretation of results. Many randomised trials in the past gave inconclusive results because a clear differnece in response rate between the treatments existed only in a subset of the study population, but the size of this fraction was not large enough to permit its statistical detection.

In view of this, we think that separate randomised trials should be carried out, in advanced NSCLC, to determine which is the best therapeutic approach in each subset of patients. Patients with only locally advanced disease should be evaluated separately, in view of both the expected better prognosis and the possible positive impact of the addition of radiotherapy. Among patients with metastatic disease, two conceptually different therapeutic strategies should be tested. Firstly, maximum effort should be devoted to increasing dose intensity and combining new drugs with different mechanisms in patients with good or intermediate performance status and age $\leqslant 70$, in view of their better tolerance to treatment and the predictable higher sensitivity of the tumour. In accordance with this consideration, in some recent trials the aggressive new combinations have been tested only in patients with performance status 0-1 (Crino' *et al.*, 1995; Langer *et al.*, 1995). Secondly, more attention should be paid to the host status in the elderly or poor performance status patients. A less toxic chemotherapy, combined with non-cytotoxic drugs

(biological response modifiers, lonidamine, melatonine, differentiating agents, etc.) could be the best therapeutic approach in these patients. This approach is probably unable to obtain a dramatic tumour shrinkage in the majority of patients; however, it may be able to delay the progression of the tumour at the price of mild toxicity, resulting in either a longer survival or a better quality of life.

### References

ARDIZZONI A, ADDAMO GF, BALDINI E, BORGHINI V, PORTALONE L, DE MARINIS F, LIONETTO R, CONTE PF, BRUZZI P, PENNUCCI MC, VENTURINI M, RINALDI M, ROSSO R AND SALVATI F. (1995). Mitomicyn + ifosfamide + cisplatin (MIP) *vs* MIP + interferon (MIP + IFN) *vs* cisplatin + carboplatin (PC): a phase II randomised trial in the treatment of advanced NSCLC. *Br. J. Cancer*, **71,** 115–119.

BONOMI PD, FINKELSTEIN DM, RUCKDESCHEL JC, BLUM RH, GREEN MD, MASON B, HAHN R, TORMEY DC, HARRIS J, COMIS R AND GLICK J. (1989). Combination chemotherapy versus single agents followed by combination chemotherapy in stage IV non-small-cell lung cancer. A study of the Eastern Cooperative Oncology Group. *J. Clin. Oncol.*, **7,** 1602–1613.

BORING CC, SQUIRES TS AND TONG T. (1992). Cancer statistics. *Cancer J. Clin.*, **42,** 19–38.

BUNN PA Jr. (1989). The expanding role of cisplatin in the treatment of non-small-cell lung cancer. *Semin. Oncol.*, **16** (Suppl 6), 1–12.

CASAGRANDE JT, PIKE MC AND SMITH PG. (1978). An improved approximate formula for calculating sample sizes, for comparing two binomial distributions. *Biometrics*, **34,** 483–486.

COMELLA P, CASARETTI R, DAPONTE A, CANNADA BARTOLI G, PARZIALE AP, PALMIERI G AND COMELLA G. (1994). Combination of vinorelbine, cisplatin, and etoposide in advanced non-small-cell lung cancer: a pilot study. *J. Chemother.*, **6,** 67–71.

COX DR. (1972). Regression models and life tables. *J. R. Stat. Soc.*, **34,** 187–220.

CRINO' L, TONATO M, DARWISH S, MEACCI ML, CORGNA E, DI COSTANZO F, BUZZI F, FORNARI G, SANTI E, BALLATORI E, SANTUCCI C AND DAVIS S. (1990). A randomized trial of three cisplatin-containing regimens in advanced non-small-cell lung cancer: a study of the Umbria Group. *Cancer Chemother. Pharmacol.*, **26,** 52–56.

CRINO' L, CLERICI M, FIGOLI F, CARLINI P, CECI G, CORTESI E, CARPI A, SANTINI A, DI COSTANZO F, BONI C, MEACCI M, CORGNA E, DARWISH S, SCARCELLA L, SANTUCCI A, BALLATORI E AND TONATO M. (1995). Chemotherapy of advanced non-small-cell lung cancer: a comparison of three active regimens. A randomized trial of the Italian Oncology Group for Clinical Research (G.O.I.R.C.). *Ann. Oncol.*, **6,** 347–353.

CRINO' L, SCAGLIOTTI G, MARANGOLO M, FIGOLI F, CLERICI M, DE MARINIS F, SALVATI F, CRUCIANI G, DOGLIOTTI L, COCCONI G, PACCAGNELLA A, ADAMO V, INCORONATO P, SCARCELLA L, MOSCONI AM AND TONATO M. (1995). Cisplatin–gemcitabine combination in non-small cell lung cancer. A phase II study. *Proc. Am. Soc. Clin. Oncol.*, **14,** 352.

CROS S, WRIGHT M, MORIMOTO M, LATASTE H, COUZINIER JP AND KRIKORIAN A. (1989). Experimental antitumor activity of navelbine. *Semin. Oncol.*, **16** (suppl. 4), 15–20.

DEPIERRE A, LEMARIE E, DABOUIS G, GARNIER G, JACOULET P AND DALPHIN JC. (1989). Efficacy of navelbine in non-small-cell lung cancer. *Semin. Oncol.*, **16** (suppl. 4), 26–29.

DONNADIEU N, PAESMANS M AND SCULIER JP. (1991). Chemotherapy of non-small cell lung cancer according to disease extent: a meta-analysis of the literature. *Lung Cancer*, **7,** 243–252.

FAULDS D. (1992). Current options in the treatment of non-small cell lung cancer. *Drugs*, **44** (suppl. 4), 46–59.

FISHER RA AND YATES F. (1963). *Statistical Tables for Biological, Agricultural and Medical Research.* Oliver & Boyd: Edinburgh.

FISHER RA. (1950). The significance of deviations from expectations in a Poisson series. *Biometrics*, **15,** 17–24.

GRILLI R, OXMAN AD AND JULIAN JA. (1993). Chemotherapy for advanced non-small-cell lung cancer: how much benefit is enough? *J. Clin. Oncol.*, **11,** 1866–1872.

HAINSWORTH J, JOHNSON D AND HANDE K. (1989). Chemotherapy of advanced non-small cell lung cancer: a randomized trial of three cisplatin-based chemotherapy regimens. *Am. J. Clin. Oncol.*, (CCT) **12,** 345–349.

IHDE DC. (1992). Chemotherapy of lung cancer. *N. Engl. J. Med.*, **327,** 1434–1441.

KAPLAN ES AND MEIER P. (1958). Non parametric estimation for incomplete observations. *J. Am. Stat. Assoc.*, **53,** 457–480.

JACOULET P, DEPIERRE A, GARNIER G AND DALPHIN JC. (1991). Study of combined navelbine-cisplatin-VP16 in the treatment of non-small lung cancer. In *Navelbine (Vinorelbine) Update and New Trends.* Pierre Fabre Oncologie (eds) pp. 171–177. John Libbey Eurotext: Montrouge.

LANGER CJ, LEIGHTON J, COMIS R, O'DWYER P, MCALEER C, BONJO C, ENGSTROM PF, LITWIN S AND OZOLS R. (1995). Paclitaxel and carboplatin in combination in the treatment of advanced non-small cell lung cancer: a phase II toxicity, response, and survival analysis. *J. Clin. Oncol.*, **13,** 1860–1870.

LE CHEVALIER T, BRISGAND D, DOUILLARD JY, PUJOL JL, ALBEROLA V, MONNIER A, RIVIERE A, LIANES P, CHOMY P, CIGOLARI S, GOTTFRIED M, RUFFIE P, PANIZO A, GASPARD MH, RAVAIOLI A, BESENVAL M, BESSON F, MARTINEZ A, BERTHAUD P AND TURSZ T. (1994). Randomized study of vinorelbine and cisplatin versus vindesine and cisplatin versus vinorelbine alone in advanced non-small-cell lung cancer: results of a European Multicenter Trial incuding 612 patients. *J. Clin. Oncol.*, **12,** 360–367.

MANTEL N. (1966). Evaluation of survival data and two new-rank order statistics arising in its consideration. *Cancer Chemother. Rep.*, **50,** 163–170.

MILLER AB, HOOGSTRATEN B, STAQUET M AND WINKEN A. (1981). Reporting results on cancer treatment. *Cancer*, **47,** 207–214.

SAKURAI M, ICHIKI M, OHASHI Y AND HAYASHI I. (1993). Phase II study of a combination regimen composed of cisplatin, carboplatin and etoposide (CPVP) in unresectable non-small cell lung cancer (NSCLC). *Proc. Am. Soc. Clin. Oncol.*, **12,** 343.

SCULIER JP, KLASTERSKY J, GINER V, BUREAU G, THIRIAUX J, DABOUIS G, EFREMIDIS A, RIES F, BERCHIER MC, SERGYSELS R, MOMMEN P AND PAESMANS M. (1994). Phase II randomized trial comparing high-dose cisplatin with moderate-dose cisplatin and carboplatin in patients with advanced non-small-cell lung cancer. *J. Clin. Oncol.*, **12,** 353–359.

THALL PF, SIMON R, ELLENBERG SS AND SHRAGER R. (1988). Optimal two-stage designs for clinical trials with binary response. *Stat. Med.*, **7,** 571–579.

TSUCHIYA S, SALTOH R, NAKANO H, MINATO K, TAKISE A, EZAWA K, FUEKI N, HOSHINO H, MAKIMOTO T, NARUSE I, NOMOTO T, TAKEI Y, ISHIHARA S, WATANABE S AND MORI M. (1993). Escalated dose carboplatin (CBDCA) with cisplatin (CDDP) + etoposide (VP16) for advanced non-small cell lung cancer (NSCLC): a phase I study. *Proc. Am. Soc. Clin. Oncol.*, **12,** 353.

WEICK JK, CROWLEY J, NATALE RB, HOM BL, RIVKIN S, COLTMAN CA JR, TAYLR SA AND LIVINGSTON RB. (1991). A randomized trial of five cisplatin-containing treatments in patients with metastatic non-small cell lung cancer: a Southwest Oncology Group Study. *J. Clin. Oncol.*, **7,** 1157–1162.

WILKINSON AND LELAND. (1988). *The System for Statistics.* Systat: Evanston, IL.

ZALCBERG JR, BISHOP JF, MILLWARD MJ, ZIMET A, LAIRD J, BARTER C, SEWARD D, MCKEAGE MJ, FRIEDLANDER ML, TONER G, BERILLE J AND BLANC C. (1995). Preliminary results of the first phase II trial of docetaxel in combination with cisplatin in patients with metastatic or locally advanced non-small cell lung cancer. *Proc. Am. Soc. Clin. Oncol.*, **14,** 351.